# Therapeutic and adverse effects of adrenaline on patients who suffer out-of-hospital cardiac arrest: a systematic review and meta-analysis

**DOI:** 10.1186/s40001-022-00974-8

**Published:** 2023-01-12

**Authors:** Hong Zhong, Zhaohui Yin, Bojin Kou, Pei Shen, Guoli He, Tingting Huang, Jing Liang, Shan Huang, Jiaming Huang, Manhong Zhou, Renli Deng

**Affiliations:** 1 Emergency Department, Affiliated Hospital of Zunyi Medical University, Zunyi, 563003 Guizhou China; 2 General Surgery Department, KweiChow Moutai Hospital, Renhuai, 564501 Guizhou China; 3 Emergency Department, KweiChow Moutai Hospital, Renhuai, 564501 Guizhou China; 4 Nursing department, Affiliated Hospital of Zunyi Medical University, Zunyi, 563003 Guizhou China

**Keywords:** Cardiac arrest, Epinephrine, Out-of-hospital, Cardiopulmonary resuscitation, Meta-analysis, Systematic review

## Abstract

**Objective:**

The efficacy and safety of epinephrine in patients with out-of-hospital cardiac arrest (OHCA) remains controversial. The meta-analysis was used to comprehensively appraise the influence of epinephrine in OHCA patients.

**Methods:**

We searched all randomized controlled and cohort studies published by PubMed, EMBASE, and Cochrane Library from the inception to August 2022 on the prognostic impact of epinephrine on patients with OHCA. Survival to discharge was the primary outcome, while the return of spontaneous circulation (ROSC) and favorable neurological outcome were secondary outcomes.

**Results:**

The meta-analysis included 18 studies involving 863,952 patients. OHCA patients with adrenaline had an observably improved chance of ROSC (RR 2.81; 95% CI 2.21–3.57; *P* = 0.001) in randomized controlled studies, but the difference in survival to discharge (RR 1.27; 95% CI 0.58–2.78; *P* = 0.55) and favorable neurological outcomes (RR 1.21; 95% CI 0.90–1.62; *P* = 0.21) between the two groups was not statistically significant. In cohort studies, the rate of ROSC (RR 1.62; 95% CI 1.14–2.30; *P* = 0.007) increased significantly with the adrenaline group, while survival to discharge (RR 0.73; 95% CI 0.55–0.98; *P* = 0.03) and favorable cerebral function (RR 0.42; 95% CI 0.30–0.58; *P* = 0.001) were lower than the non-adrenaline group.

**Conclusion:**

We found that both the randomized controlled trials (RCTs) and cohort studies showed that adrenaline increased ROSC in OHCA patients. However, they were unable to agree on a long-term prognosis. The cohort studies showed that adrenaline had an adverse effect on the long-term prognosis of OHCA patients (discharge survival rate and good neurological prognosis), but adrenaline had no adverse effect in the RCTs. In addition to the differences in research methods, there are also some potential confounding factors in the included studies. Therefore, more high-quality studies are needed to fully confirm the effect of adrenaline on the long-term results of OHCA.

## Introduction

Worldwide, out-of-hospital cardiac arrest (OHCA) is still one of the main causes of death [1]. The key to successful cardiopulmonary resuscitation (CPR) after cardiac arrest (CA) first depends on the time of CPR initiation. Meanwhile, effective coronary perfusion and timely restoration of myocardial blood supply also play important roles in succeeding CPR. Epinephrine has been the first choice of medicine with stimulating effects on α and β receptors in treating CA since the 1960s [2]. During CPR, the activation of α-adrenergic receptors can increase aortic diastolic pressure and myocardial blood flow. Numerous studies indicated that epinephrine increased the rate of return of spontaneous circulation (ROSC) by the activation of its alpha receptors [3, 4].

However, epinephrine increases cardiac output, while the activation of its β-adrenergic leads to dysrhythmias and increases myocardial oxygen demand [5]. It has been reported that although epinephrine improves ROSC rates, it does not improve survival to hospital discharge or favorable neurologic outcomes [6, 7]. Some studies also suggested that epinephrine does not improve survival to hospital discharge. On the contrary, it even causes deterioration in neurological function. According to a randomized controlled study by Perkins et al. [8], a higher rate of survival to hospital discharge was observed in the epinephrine group compared with the placebo group, but among patients who survived at discharge, the incidence of brain injury in the adrenaline group nearly doubled compared with the placebo group, and other outcomes such as survived to hospital discharge with a favorable neurologic outcome, survival at 3 months, or neurologic outcomes at 3 months were no significant differences between the two groups. It can be noticed that epinephrine may be unprofitable or even harmful. Although epinephrine has been widely used to treat OHCA, its beneficial effect remains controversial [9–11].

Recent meta-analysis results such as Kempton et al. indicated that the epinephrine group was better than the placebo group in ROSC rate and survival to hospital admission. Survival to hospital discharge and neurological status between the two groups were not significantly different. However, this study included only five randomized controlled studies [12]. Vargas et al. [13] included 15 randomized controlled trials (RCTs) in their meta-analysis. Standard dose of epinephrine was compared with placebo, high doses of epinephrine, and vasopressin. The results showed that CA patients' survival rates and outcomes improved when epinephrine was administered, but there was no subgroup analysis on different initial cardiac rhythms included. Based on a recent meta-analysis, epinephrine improved ROSC rates and survival rate of leave hospital in CA patients but worsened the neurological prognosis of OHCA patients [14]. The original literature included in this meta-analysis did not limit the population characteristics, but contained CA patients both in-hospital and out-of-hospital into the study; Prognostic factors, such as age, co-morbidity, etiology, and initial cardiac rhythms, were different in patients with in-hospital cardiac arrest (IHCA) and OHCA. Compared with OHCA patients, the time from CA starts to the point when CPR is performed is shorter, same as the duration of defibrillation, the time of administration of medicine in patients who suffered from CA in the hospital. It might affect the effect of epinephrine administration during resuscitation. Consequently, epinephrine's impact on the outcome of patients with OHCA needs to be evaluated again through systematic reviews and meta-analyses. The meta-analysis only included the results of RCTs and cohort studies of OHCA patients, and divided the initial cardiac rhythms of OHCA patients into shockable rhythms and non-shockable rhythms subgroups to sufficiently illustrate the effect of epinephrine.

## Methods

### Type of studies

RCTs and cohort studies on the effect of epinephrine on outcomes in patients with OHCA were included in this meta-analysis. The studies only involved adult patients with OHCA, and the outcome indicators included the rate of ROSC, survival to hospital discharge, and favorable neurologic status at discharge.

### Study eligibility and exclusion criteria

#### Study eligibility

(1) Study patients: non-traumatic OHCA patients (age ≥ 14 years). (2) Intervention types: epinephrine versus no epinephrine (placebo), and the mode of administration is intravenous (IV) or intraosseous (IO). (3) Outcome measures: the survival rate at discharge which was defined as the survival rate from survival to discharge or the 30 day survival rate was the primary outcome; the secondary outcomes included ROSC rate and favorable neurological outcomes. At least one of the aforementioned outcome indicators must be present in the included studies. (4) Study type: randomized controlled study or cohort study.

#### Exclusion criteria

(1) Studies without a control group or ones that do not meet the requirements of an RCT or cohort study. (2) Patients with traumatic IHCA (age < 14 years). (3) Studies in which epinephrine was mainly given by intracardiac injection or through an endotracheal tube were excluded for the doses, pharmacokinetics, and pharmacodynamics were different. (4) The original text was not obtained in various ways, and there was not enough information. (5) Data from the original study were not able to be transformed and used in this study. (6) For the repeatedly published literature, we selected the one with the most complete data to avoid repeated quotations.

### Retrieval strategy

We comprehensively searched three databases (PubMed, EMBASE, and the Cochrane Library) for RCTs or cohort studies from the inception of the databases to August 2022 without restriction on language. Here are the keywords or medical subject headings (MeSH) terms used: “epinephrine”, “adrenaline”, “heart arrest”, “cardiopulmonary arrest”, “Pulseless electrical activity”, and “Ventricular fibrillation”.

### Data extraction

The data were extracted by two researchers independently searching three databases, and any disagreement was resolved by discussion. The following information was collected in each eligible study: authors, country, publication year, study design, intervention measures, etc. Indicators of outcome were as follows: survival rate at discharge was the primary outcome, and ROSC rate and a beneficial neurological outcome were secondary outcomes. A beneficial neurological status was defined as 1 or 2 points of a CPC score [15] or a score of 3 or less on the MRS[16] or a GCS of 14 or 15 [17].

### Literature quality assessment

The studies included in this article are mainly RCTs and cohort studies. Two researchers independently evaluated the quality of the literature included based on the bias risk evaluation criteria of the Cochrane collaborative network and the Newcastle-Ottawa scale (NOS). Disagreements among reviewers were settled through discussion. The quality of RCTs was assessed by the Cochrane Collaboration RCT risk assessment tool, which assessed the biases of seven entries that included random sequence generation, allocation concealment, blinding of participants and personnel, blinding of outcome assessment, incomplete outcome data, selective reporting, and other biases. The NOS scale was used to appraise the outcome of cohort studies, comprising the cohort selection, group comparability, and outcomes, with a total score of nine points.

### Statistical analysis

For statistical evaluations, Review Manager Version 5.3 was used. The results were compared by relative risk (RR) and 95% confidence interval (95% CI). The *Q*-value test and *I*^2^ test were used to test heterogeneity (*I*^2^ > 50% or *P* < 0.05, indicating substantive heterogeneity) [18]. Based on the heterogeneity in the population characteristics of such studies, a meta-analytical report based on the random-effects model was conducted, and the results obtained were more conservative than the fixed-effects model. Given the obvious correlation between the initial cardiac rhythms’ type of OHCA patients and the treatment effect and prognosis [19]. This meta-analysis further took the initial rhythms of OHCA patients with initial shockable rhythms, including ventricular tachycardia and ventricular fibrillation (VT or VF), and non-shockable rhythms, including pulseless electrical activity (PEA) and ventricular arrest as a subgroup analysis. For publication bias in the literature, we applied the rank correlation test and linear regression to quantitatively evaluate the symmetry of the funnel plot, and when the test result was *P* < 0.05, there are statistically significant in publication bias.

## Results

**Fig. 1:**
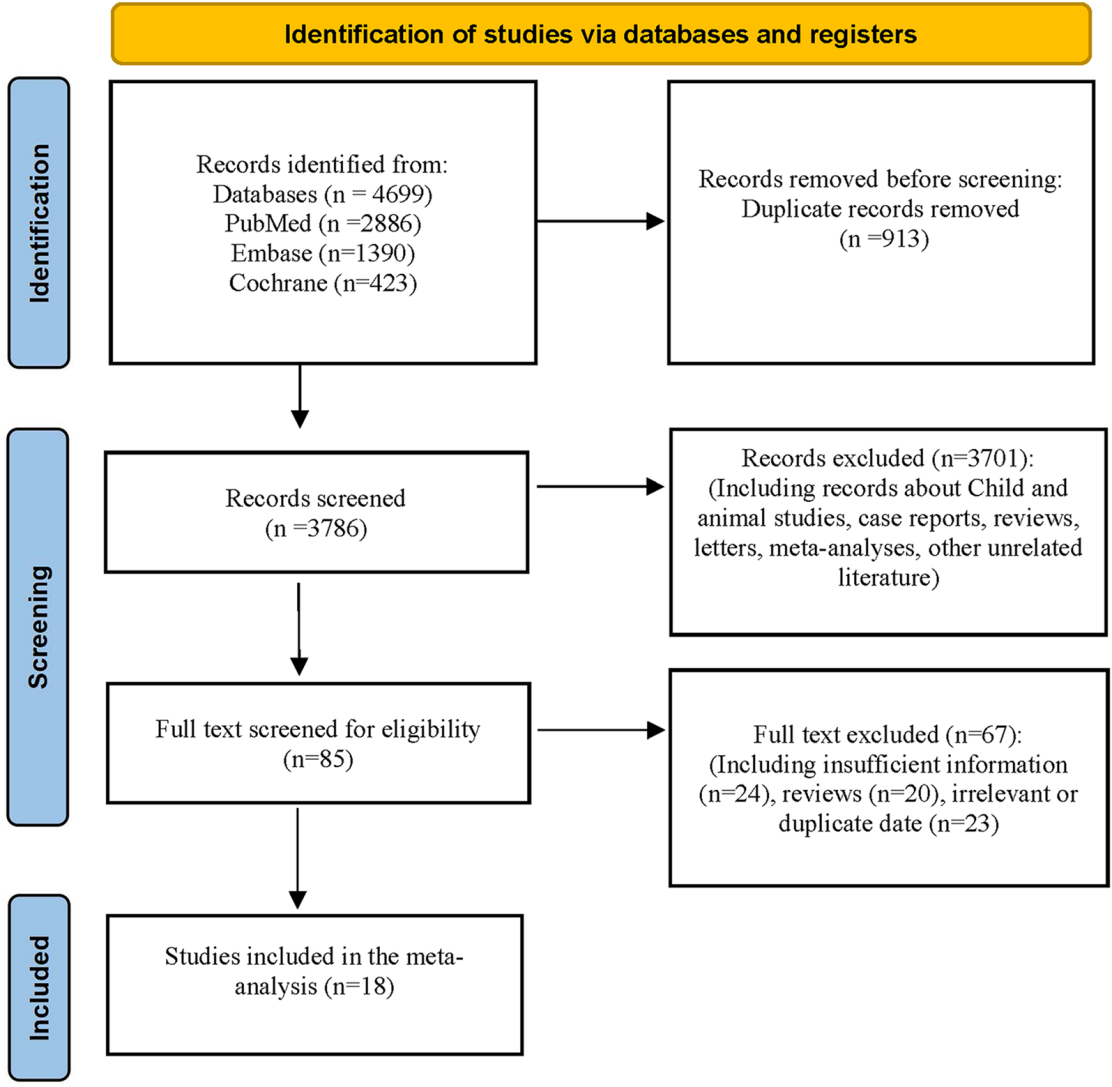
The selection process of studies

We searched 4699 studies from PubMed, EMBASE, and Cochrane library first, and excluded 913 repetitive literatures. After reading the title and abstract, 3701 articles were removed based on the inclusion and exclusion criteria. 85 studies underwent full-text review. Then, we excluded 67 studies by reading the full text. Finally, 18 articles were included, involving 3 RCTs [8, 20, 21] and 15 cohort studies [3, 6, 16, 17, 22–31]. The document screening process is illustrated in Fig. 1.

In Table 1, we summarized the basic characteristics of the included studies**.** Among the included studies, 15 were cohort studies and 3 were randomized controlled studies. There were 863,952 patients included in the study, of which 127,178 were treated with adrenaline and 736,774 were not. A total of 16 studies [3, 8, 16, 17, 20–28, 30–32] comprised patients with shockable or non-shockable rhythms, and only 2 studies [6, 29] had no records of the patient's initial cardiac rhythms. Among 3 randomized controlled studies [8, 20, 21], all of them reported ROSC and survival to discharge, 2 studies [8, 21] reported the neurologic outcomes at discharge, and the survival rates and neurologic outcomes at 3 months were reported in only one study [8]. In 15 cohort studies, all of them but one study [30] reported survival to discharge, 13 studies [3, 6, 16, 17, 22–27, 29, 31, 32] reported ROSC, and 12 studies [3, 6, 16, 17, 22–24, 26–28, 30–32] reported the favorable neurological status, and only 1 study [31] reported 1-year survival rate. In all studies, a standard dose of adrenaline administration was compared with no adrenaline administration or placebo.

**Table 1:** **C**haracteristics of included studies

Study	Country	Design	Total number of people (*n*)		Initial cardiac rhythm (*n*)		Outcome indicators	Study	Country	Design	Total number of people (*n*)		Initial cardiac rhythm (*n*)		Outcome indicators
			Adrenaline group	Non-adrenaline group	Shockable	Non- Shockable					Adrenaline group	Non-adrenaline group	Shockable	Non- shockable	
Jacobs 2011	Australia	RCT	272	262	245	289	ROSC; survival to discharge; CPC at hospital discharge (CPC<3)	Baert 2020	France	Cohort study	24294	2714	3448	23560	Survival at D30; ROSC; the neurological outcome at D30
Machida 2012	Japan	Cohort study	49	443	75	416	ROSC: survival to hospital discharge; good neurologic recovery at hospital discharge	Dumas 2014	France	Cohort study	1134	422	845	N/A	Favorable neurological outcome at discharge (CPC<3)
Perkins 2018	England	RCT	4015	3999	1445	6221	ROSC; survival at hospital discharge and at 3 months, and the neurologic outcomes at hospital discharge and at 3 months (MRS≤3)	Fukuda 2015	Japan	Cohort study	770	6301	845	N/A	Favorable neurological status at 1 month; 1-month survival; ROSC
Kaji 2014	America	Cohort study	160	24	66	N/A	ROSC; survival to hospital discharge; survival with GCS of 14 or 15	Fukuda 2016	Japan	Cohort study	33400	33400	8040	58760	ROSC; 1-month survival; neurological status one month
Hayakawa 2013	Japan	Cohort study	318	315	173	460	ROSC; favorable neurological outcome 30 days; survival of 30 days	Nordseth 2012	Sweden	RCT	101	73	N/A	174	ROSC; survival to discharge
Goto 2013	Japan	Cohort study	23676	185901	15494	194085	ROSC; survival at 1 month; survival at 1 month with favorable neurological outcome (defined as a CPC score of 1 or 2)	Hagihara 2012	Japan	Cohort study	15030	73	31157	386024	ROSC; survival at 1 month; 1-month survival with CPC category 1 or 2
Neset 2013	Sweden	Cohort study	119	104	187	36	Survival to hospital discharge	Yu Wang 2022	China	Cohort study	572	961	N/A	N/A	ROSC; survival to hospital discharge
Olasveengen 2012	Sweden	Cohort study	367	481	284	564	ROSC; survival to hospital discharge; neurologic outcome at hospital discharge	Hayashi 2012	Japan	Cohort study	1013	2148	506	2655	ROSC; 1-month survival; neurologically intact 1-month survival
								Nakahara 2013	Japan	Cohort study	13421	82658	14943	81136	Survival and neurologically intact survival with the Glasgow–Pittsburgh cerebral performance category score 1-2 at one month or at discharge
								2021 Matsuyama	Japan	Cohort study	8467	14410	N/A	N/A	1-month survival; ROSC; 1-month survival with favorable neurological outcome

*RCT* randomized controlled trial, *N/A* not available, *ROSC* return of spontaneous circulation, *CPC* Cerebral Performance Category, *MRS* Modified Rankin Scale, *GCS* Glasgow Coma Score

### Quality assessment

**Fig. 2:**
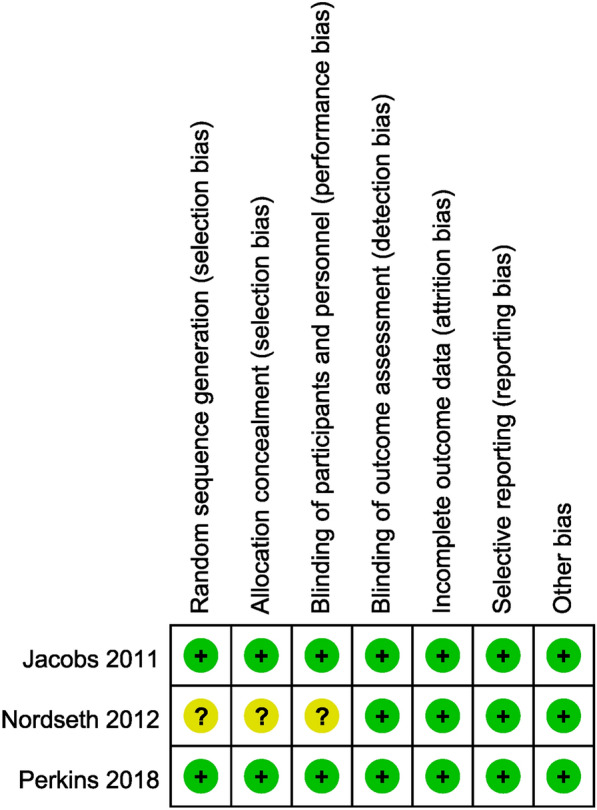
Assessment of bias in all included RCTs. Low, high, and unclear risk of bias were indicated by green, red, and yellow circles, respectively

We used the Cochrane Collaboration risk assessment tool to assess the quality of RCTs and summarized the potential sources of bias in RCTs. The results are presented in Table 2 and illustrated in Fig. 2. Two RCTs were endowed with “low risk of bias” and only one RCT was assessed as having an “unclear risk of bias” for at least one domain, and “high bias risk” was not assessed for any study. The bias risk of 15 cohort studies was assessed using the Newcastle-Ottawa Scale (Table 3), and most of them were considered high quality.

**Table 2:** Cochrane risk bias assessment tool for RCTs

Study	Allocation generation	Allocation concealment	Blinding of participants	Blinding of assessors	Outcome complete	Outcome selective	Other biases
2011 Jacobs	Low	Low	Low	Low	Low	Low	Low
2012 Nordseth	Unclear	Unclear	Unclear	Low	Low	Low	Low
2018 Perkins	Low	Low	Low	Low	Low	Low	Low

**Table 3:** Newcastle–Ottawa quality assessment scale for the cohort studies

Study	Selection of cohort (4)				Comparability (1)	Outcome (3)		
	Representativeness of the exposed cohort	Selection of the non-exposed cohort	Ascertainment of exposure	Demonstration that outcome of interest was not present at start of study	Comparability of cohorts on the basis of the design or analysis	Assessment of outcome	Was follow-up long enough for outcomes to occur	Adequacy of follow-up of cohorts
2015 Fukuda	–	⭐	⭐	⭐	⭐	–	⭐	⭐
2016 Fukuda	⭐	⭐	⭐	⭐	⭐	–	⭐	–
2012 Machida	⭐	⭐	⭐	⭐	⭐	⭐	⭐	⭐
2012 Hagihara	⭐	⭐	⭐	⭐	⭐	–	⭐	⭐
2012 Hayashi	⭐	⭐	⭐	⭐	⭐	⭐	⭐	⭐
2012 Olasveengen	⭐	⭐	⭐	⭐	⭐	⭐	⭐	⭐
2013 Goto	⭐	⭐	⭐	⭐	⭐	⭐	⭐	⭐
2013 Neset	⭐	⭐	⭐	⭐	–	⭐	⭐	⭐
2014 Kaji	⭐	⭐	⭐	⭐	⭐	⭐	⭐	⭐
2014 Dumas	⭐	⭐	⭐	⭐	⭐	–	⭐	⭐
2021 Matsuyama	⭐	⭐	⭐	⭐	⭐	⭐	⭐	⭐
2013 Hayakawa	⭐	⭐	⭐	⭐	⭐	–	⭐	⭐
2020 Baert	⭐	⭐	⭐	⭐	⭐	–	⭐	⭐
2013 Nakahara	⭐	⭐	⭐	⭐	⭐	⭐	⭐	⭐
2022 Yu Wang	⭐	⭐	⭐	⭐	⭐	–	⭐	⭐

For each numbered item in the selection and exposure categories, the study may receive a maximum of one star. For comparability category, up to two stars may be given

### Return of spontaneous circulation

**Fig. 3:**
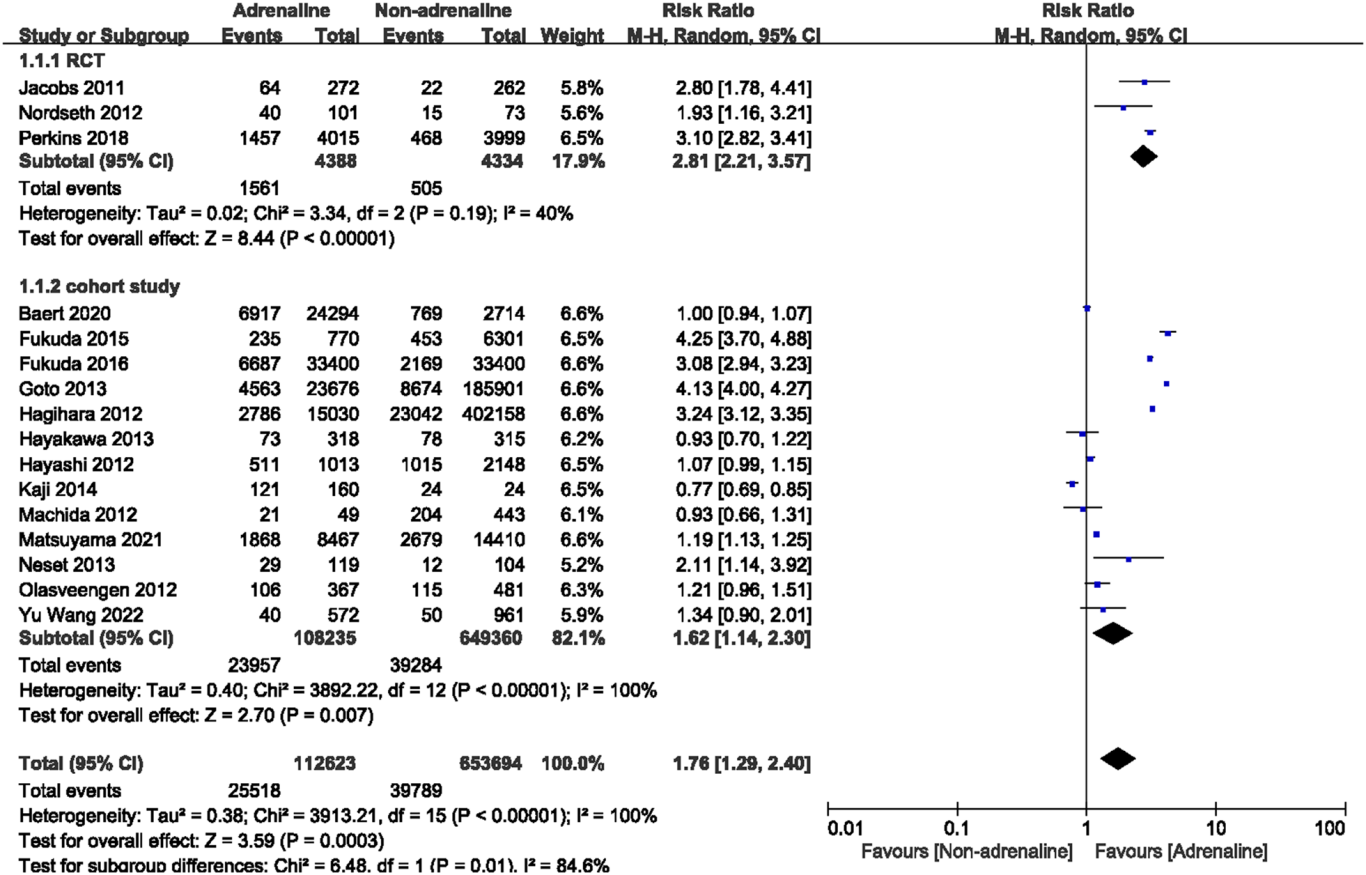
Forest plot of the effects of adrenaline versus non-adrenaline on ROSC

ROSC rates were reported in 16 studies (3 RCTs and 13 cohort studies) with a total of 766,317 cases, including 112,623 patients who used adrenaline and 653,694 patients without the use of adrenaline. Since the heterogeneity test results displayed that *I*^2^ was 40% and 100% for the RCTs and cohort studies, respectively, substantial differences were considered to exist among the studies. Therefore, we used the random-effects model to conduct statistical analysis on these outcome measures. As a result, the ROSC rate in the adrenaline group was higher than that in the adrenaline-free group [RCTs: RR = 2.81, 95% CI (2.21, 3.57), *P* = 0.001; cohort study: RR = 1.62, 95% CI (1.14, 2.30), *P* = 0.007] (Fig. 3).

**Fig. 4:**
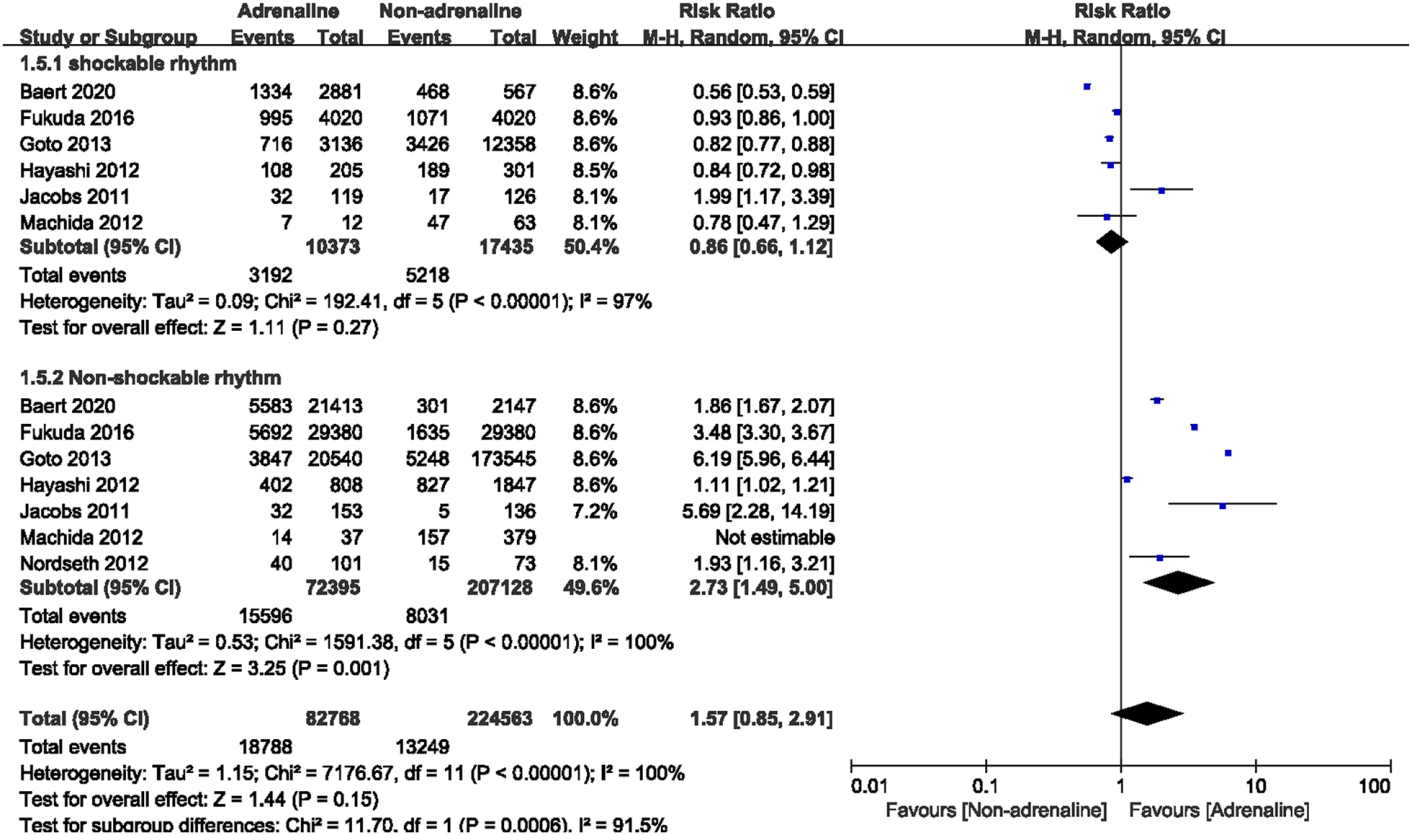
Forest plot of subgroup analysis on ROSC

A subgroup analysis was performed according to initial cardiac rhythms, in which 6 studies recorded a total of 27,808 OHCA patients with shockable rhythms, including 10,373 in the epinephrine group and 17,435 in the non-epinephrine group. Heterogeneity existed among these studies (*I*^2^ = 97%). In combination with the random-effects model, ROSC rates were no significant differences between the two groups. [RR = 0.86, 95% CI (0.66–1.12), *P* = 0.27] (Fig. 4). A total of 279,523 OHCA patients with non-shockable rhythms were recorded in 7 studies. It included 72,395 patients who used epinephrine and 207,128 patients who did not use epinephrine. There was heterogeneity among studies (*I*^2^ = 100%), and after combining by random-effects model, we found that epinephrine increased the ROSC rates in OHCA patients with non-shockable rhythms. [RR = 2.73, 95% CI (1.49–5.00), *P* = 0.001]. The forest plot is shown in Fig. 4.

### Survival to hospital discharge

**Fig. 5:**
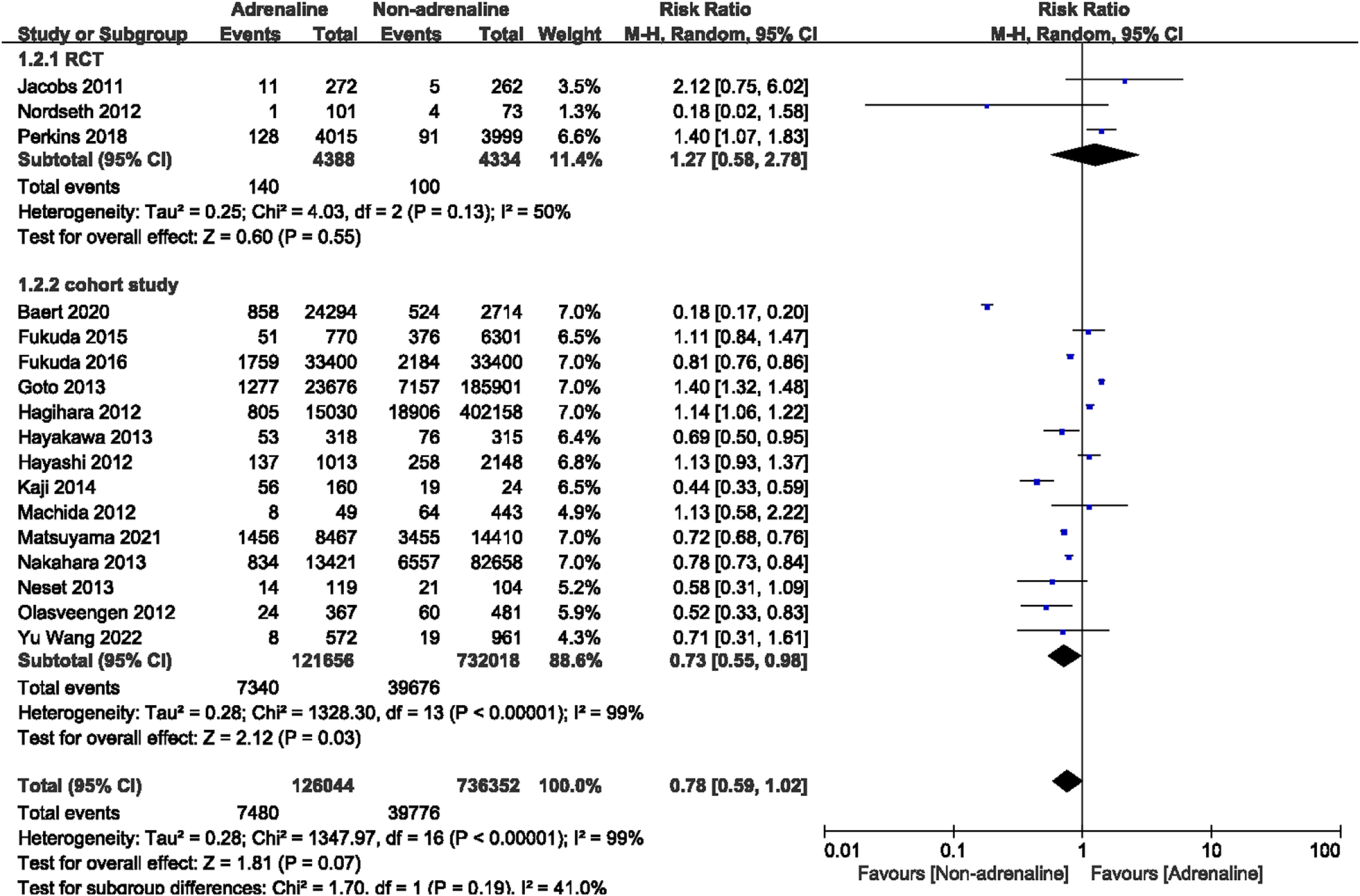
Forest plot of the effects of adrenaline versus non-adrenaline on survival to hospital discharge

Seventeen studies (3 RCTs and 14 cohort studies) reported the survival rate at discharge, with a total of 862,396 cases, including 126,044 cases with adrenaline and 736,352 cases without the use of adrenaline. The results of the heterogeneity test indicated that *I*^2^ was 50% in RCTs and 99% in cohort studies. The statistical analysis of this observation index using the random-effects model showed that in the RCTs, no substantial differences existed in survival to hospital discharge between the two groups. However, we found that in the cohort studies, a higher rate of survival at discharge appeared in the non-epinephrine group. [RCT: RR = 1.27, 95% CI (0.58, 2.78), *P* = 0.55; cohort study: RR = 0.73, 95% CI (0.55, 0.98), *P* = 0.03] (Fig. 5).

**Fig. 6:**
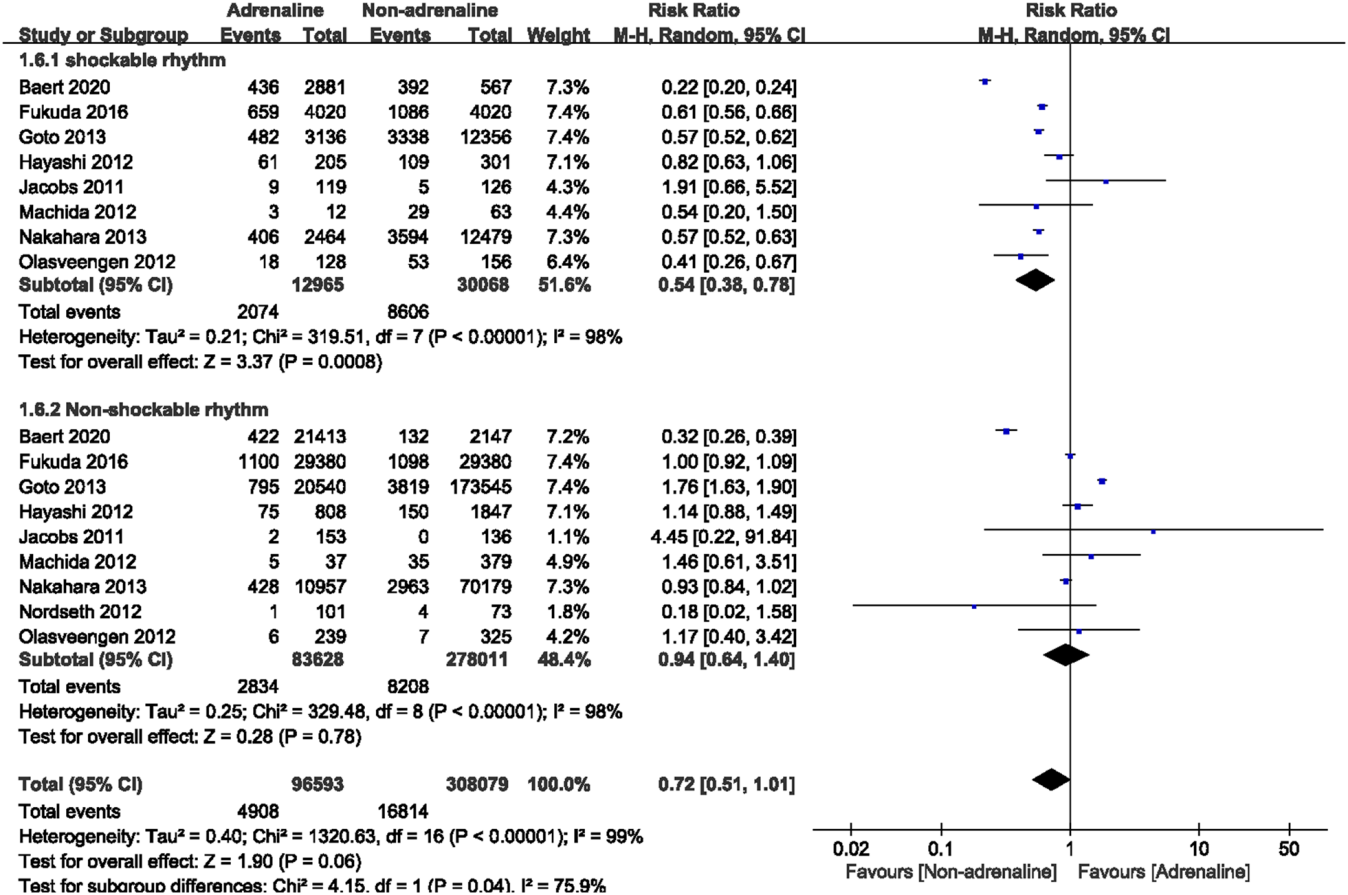
Forest plot of subgroup analysis on survival to discharge

Based on the initial cardiac rhythms, subgroup analyses were conducted. Eight articles recorded 43,033 OHCA patients with shockable rhythms, and the adrenaline group included 12,965 patients, while the non-adrenaline group included 30,068 patients. Among the studies, heterogeneity existed (*I*^2^ = 98%). When combined with a random-effects model, we found improved survival at discharge in OHCA patients with shockable rhythms in the absence of epinephrine [RR = 0.54, 95% CI (0.38–0.78), *P* = 0.0008] (Fig. 6). A total of 361,639 OHCA patients with non-shockable rhythms were recorded in nine articles, including 83,628 patients with adrenaline and 278,011 patients without adrenaline. Heterogeneity existed among the studies (*I*^2^ = 98%). After combining with the random-effects model, no significant differences in survival to hospital discharge were observed between the two groups in OHCA patients with non-shockable rhythms. [RR = 0.94, 95% CI (0.64–1.40), *P* = 0.78] (Fig. 6).

### Favorable neurological outcomes

**Fig. 7:**
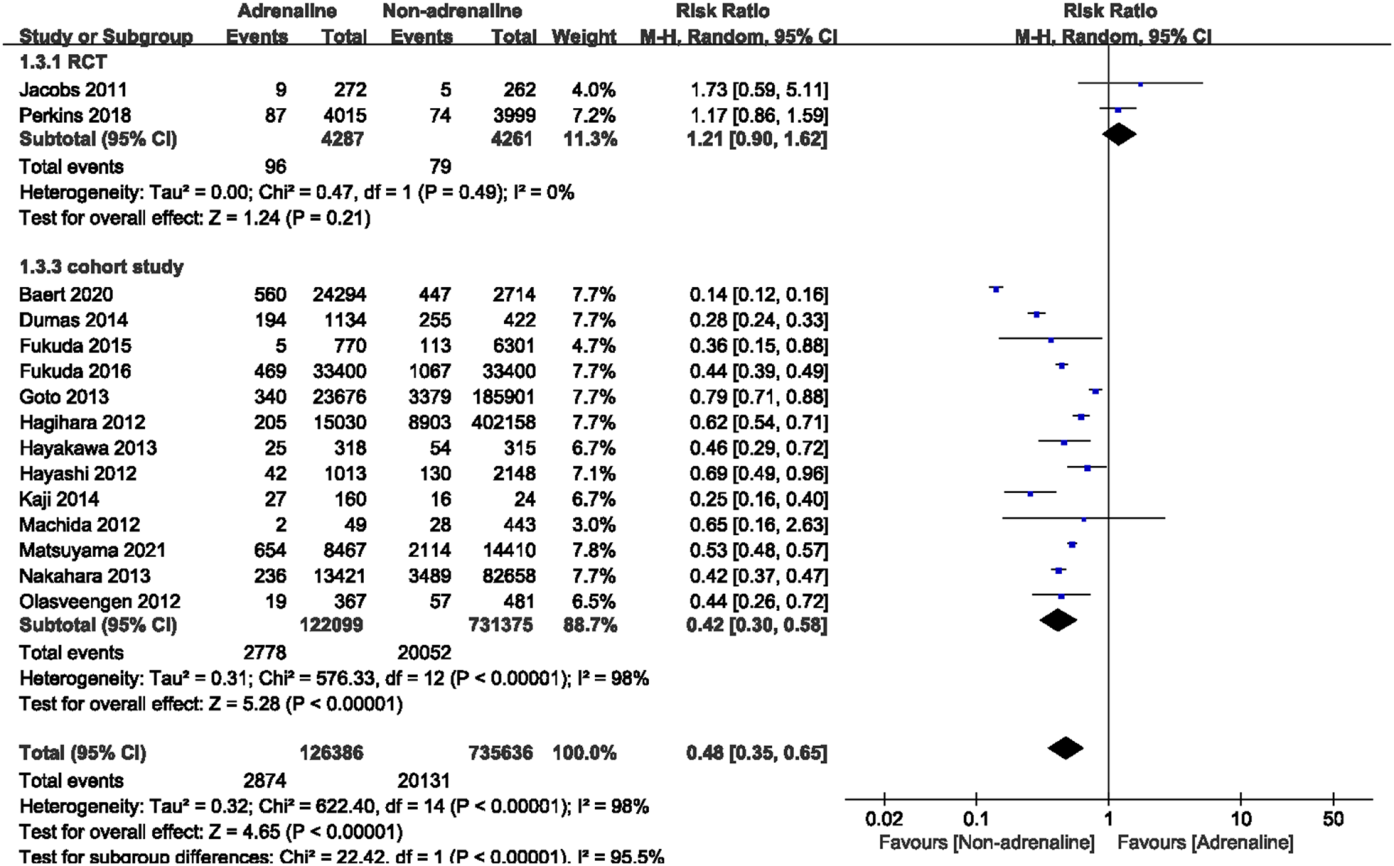
Forest plot of the effects of adrenaline versus non-adrenaline on neurological outcomes

Fifteen studies (2 RCTs and 13 cohort studies) reported favorable neurological outcomes, with a total of 862,022 cases, including 126,386 cases in that adrenaline was administered and 735,636 cases that did not use adrenaline. The heterogeneity test results indicated that I^2^ was 0% in RCTs and 98% in cohort studies. Statistical analysis of this observation index was conducted using a random effect model. Neither group showed a difference in the results of RCTs in terms of good neurological prognoses. In cohort studies, using adrenaline was detrimental to the neurological prognoses of OHCA patients. [RCT: RR = 1.21, 95% CI (0.90, 1.62), *P* = 0.21; cohort study: RR = 0.42, 95% CI (0.30, 0.58), *P* = 0.001] (Fig. 7).

**Fig. 8:**
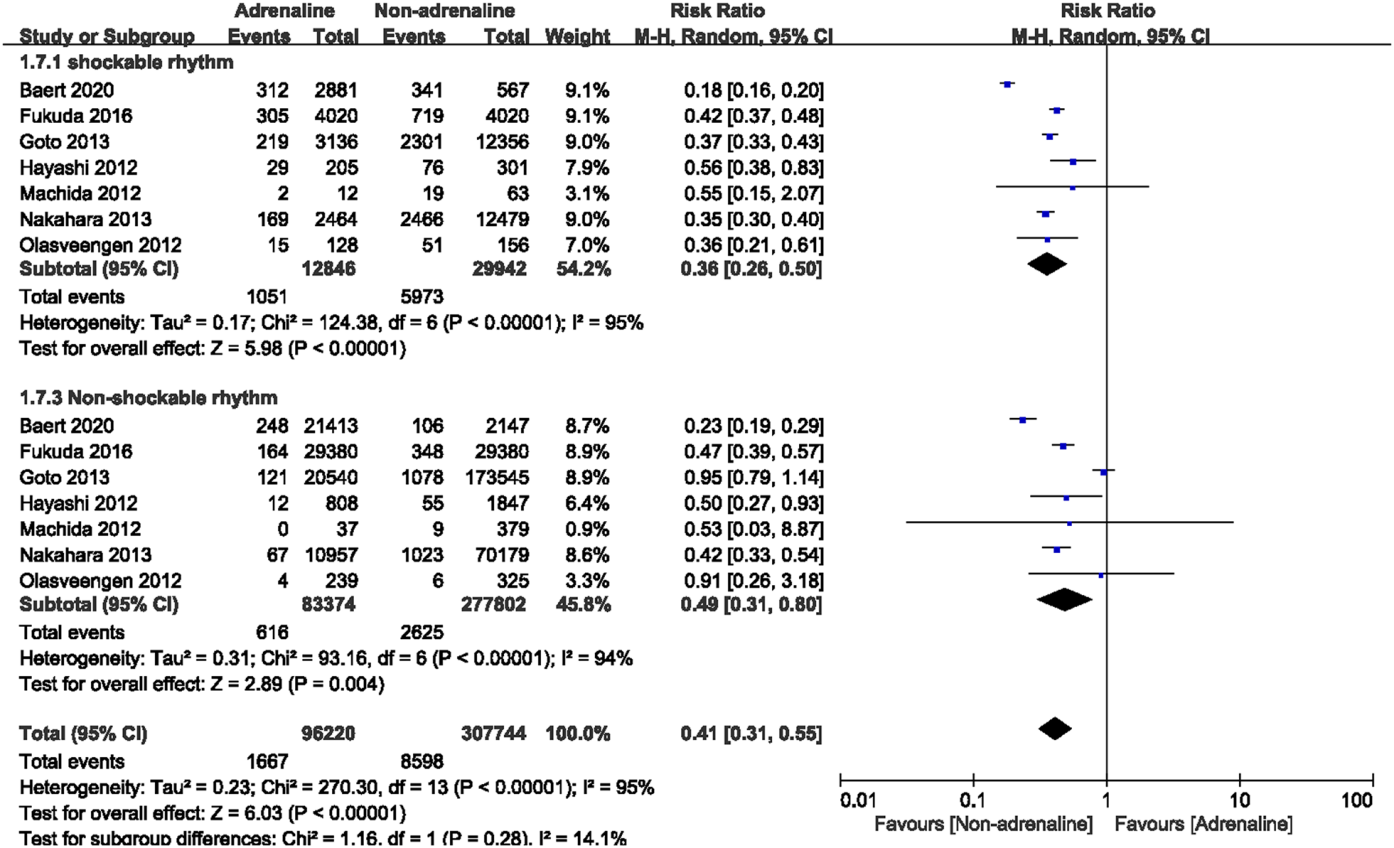
Forest plot of subgroup analysis on favorable neurological outcomes

For subgroup analysis by initial cardiac rhythms, a total of 42,788 OHCA patients with shockable rhythms were recorded in 7 studies, including 12,846 cases in that epinephrine was administered and 29,942 cases that were not. There was heterogeneity between studies (*I*^2^ = 95%), and after combining with the random effect model, we found a worse neurological prognosis appeared in the adrenaline group. [RR = 0.36, 95% CI (0.26–0.50), *P* = 0.001] (Fig. 8); seven studies documented a total of 361,176 OHCA patients with non-shockable rhythms, including 83,374 patients who used epinephrine and 277,802 who did not. Significant heterogeneity was observed between studies (*I*^2^ = 94%), so a random-effects model was used to analyze the outcome indicators. The results indicated that OHCA patients with non-shockable rhythms who were not given epinephrine had a better neurological prognosis [RR = 0.49, 95% CI (0.31–0.80), *P* = 0.004] (Fig. 8).

### Literature publication bias

We applied the rank correlation test and linear regression method to draw the funnel plots of ROSC, survival at discharge, and good neurological outcomes of OHCA patients in the adrenaline group and non-adrenaline group. As shown in the results, there is no publication bias (*P* > 0.05) for all outcome measures [Begg’s test value (*P* = 0.773) and Egger’s test value (*P* = 0.173) of ROSC; Begg’s test (*P* = 0.820) and Egger’s test (*P* = 0.736) of survival to discharge; Begg’s test (*P* = 0.767) and Egger’s test (*P* = 0.589) of the favorable neurological outcomes].

## Discussion

Based on the latest literature, our systematic review and meta-analysis were used to appraise whether epinephrine is effective and safe during the resuscitation of OHCA patients. The results of RCTs and cohort studies indicated that epinephrine increased the rate of ROSC in OHCA patients. The underlying mechanism could be that epinephrine can mediate the contraction of small arteries to increase aortic diastolic pressure by activating α-adrenergic receptors, thereby increasing coronary artery blood flow, preferentially delivering blood to the heart and brain, and increasing the probability of ROSC [33, 34]. This finding is consistent with the meta-analyses by Loomba et al. [35] and Morales et al. [36] that epinephrine increased the rate of ROSC in patients with OHCA.

Although adrenaline is known to increase the ROSC rate in OHCA patients, whether it is beneficial to the survival rate at discharge and favorable neurological outcomes in OHCA patients remains debatable. In this article, the results of RCTs showed that whether adrenaline was used or not had no effect on the survival rate at discharge and good neurological status of OHCA patients, which was consistent with the meta-analysis results of Lin et al. [37] Therefore, why did not the survival rate at discharge and good neurological prognoses increase as the incidence of ROSC in patients receiving epinephrine treatment? The reasons are uncertain, it may be that although epinephrine can promote ROSC by increasing coronary blood flow and cerebral perfusion through its powerful α-adrenergic effects [38], β-adrenergic effects can also lead to arrhythmias and increased myocardial oxygen demand, and even recurrent cardiac arrest [5]. Additionally, platelets are activated by α-adrenergic stimulation, thereby promoting thrombosis [39] and impairing microvascular blood flow in the brain. Thus, cerebral ischemia becomes more severe during CPR and after ROSC [40]. The brain is extremely sensitive to cerebral ischemia and reperfusion injury and has a poor ability to regain functionality than hearts and other organs after circulation is restored [41]. The cohort studies showed that adrenaline not only reduced the survival rate at discharge but also worsened neurological outcomes. A study published by Ong et al. [9] that compared OHCA patients who were treated with adrenaline and those who were not found that the duration of CPR was an important confounding factor affecting the outcome of adrenaline on OHCA patients. For example, due to early ROSC, adrenaline treatment was not given to some patients, and these patients often had better neurological prognoses. However, in our meta-analysis, the majority of original studies included did not record the duration of CPR or have inaccurate records, so no relevant subgroups were set up to analyze the impact of the duration of CPR on adrenaline.

The current CPR guidelines recommend that 1 mg of adrenaline, a standard dose of it, is given every 3–5 min during CPR, but the total cumulative dose is not mentioned [42]. Fukuda et al. [24] showed that repeated administration of epinephrine is harmful. Although the optimal dose of epinephrine is still unclear, increasing the cumulative dose of epinephrine may worsen the survival rate and neurological prognoses of patients with OHCA [30, 43]. This is because repeated administration of epinephrine indicates that the time required for resuscitation will be longer and the adverse reaction of epinephrine after resuscitation may be stronger. As for the timing of epinephrine administration, Hayakawa et al. [26] showed that in OHCA patients, neurological prognoses in 30 days may improve when it was administered early after CA. The frequency of positive neurological outcomes increased by 1.1 times when the epinephrine was administered 1 min earlier. Hayashi et al. [27] found that only when using adrenaline within 10 min of CA can increase the survival rate of patients and improve neurological outcomes, but it is clinically very difficult to give adrenaline in this early period and only a few patients received intravenous epinephrine within 10 min after the occurrence of OHCA. These kinds of OHCA patients are needed to better determine the impact of early use of adrenaline.

RCTs and cohort studies failed to reach an agreement on survival to discharge and good neurological prognosis. In addition to the different nature of the two study methods, it is also possible that most of the original literature included did not record the administration time, dosage, CPR time, and other confounding factors, which is also the reason for the high heterogeneity of this meta-analysis. In addition, there are also some differences in the treatment of OHCA patients after hospital admission, and these differences are often not reported or difficult to control. Good neurological outcomes, together with health-related quality of life and survival, were ranked as the most important outcomes. If the chance of rehabilitation is very small or the risk of nervous system damage is high, which leads to a decrease in the quality of life after rehabilitation, some patients may not be willing to receive heavy treatment [44]. Therefore, the outcomes of OHCA patients may be greatly affected when they choose to leave the hospital after discontinuing treatment without medical treatment such as target temperature management [45] or percutaneous coronary intervention [46].

Only two studies reported long-term outcomes. Perkins et al. [8] pointed out that although epinephrine can increase the 3-month survival rate of OHCA patients, there is no improvement in neurological prognoses. Olasveengen et al. [31] reported that adrenaline reduced the 1-year survival rate of OHCA patients. However, the above results are not convincing enough. We still need more well-designed trials to appraise the long-term outcomes of OHCA patients after using adrenaline. In summary, we should reconsider the use of adrenaline. In future studies, confounding factors, such as the administration time, duration of CPR, hospitalization treatment methods, and dosage regimen, should be recorded in detail. Therefore, we can control these potential confounding factors, and the effectiveness and safety of adrenaline on OHCA patients can be fully confirmed.

Should OHCA patients with different initial rhythms be treated differently? Subgroup analysis showed that epinephrine decreased the survival rate at discharge and worsened the neurological prognoses of OHCA patients with shockable rhythms; however, no effects were observed on ROSC between patients who used epinephrine and those who did not. In contrast, among OHCA patients with non-shockable rhythms, epinephrine increased ROSC rates but worsened neurological prognoses, and has no effect on survival to hospital discharge. It was suggested by the latest CPR guideline that using epinephrine after initial defibrillation failure could be appropriate for CA patients with shockable rhythms [42]. Andersen et al.’s [47] study on CA patients with shockable rhythms indicated that the worst prognosis at discharge could be observed when epinephrine was administered within 2 min of the first defibrillation. There is a possibility that early use of adrenaline may interfere with some necessary steps, such as high-quality chest compressions, airway support, and defibrillation, but the characteristics and resuscitation strategies of in-hospital CA patients differ from those of OHCA patients, so the applicability of the observed results to the out-of-hospital setting remains uncertain. Therefore, administering epinephrine at the right time is particularly important. Further research is needed to determine the best resuscitation strategy for OHCA patients with different initial rhythms.

However, there are some weaknesses in our study. First, most studies included in this meta-analysis are observational studies, which makes it difficult to adjust confounding factors, such as administration time of adrenaline, CPR quality, etc. Second, CPR treatment should be individualized according to the etiology and progression of the patient. Consequently, it is difficult to implement a strictly standardized intervention protocol between the trial and control groups. And there are also differences in the dosage and duration of epinephrine treatment in different studies. Furthermore, the included studies may have potential confounding factors, such as CA time, bystander CPR, the response time of emergency medical service of various countries, differences in-hospital treatment, and rescue experience of medical staff, etc. The final results and the credibility of the research will be affected by the factors above, and they are also the cause of heterogeneity in outcome indicators.

## Conclusion

In summary, this meta-analysis found that both RCTs and cohort studies showed that adrenaline increased ROSC in OHCA patients. However, they were unable to agree on a long-term prognosis. The cohort studies showed that adrenaline had an adverse effect on the long-term prognosis of OHCA patients (discharge survival rate and good neurological prognosis), but adrenaline had no adverse effect in the RCTs. In addition to the differences in research methods, there are also some potential confounding factors in the included studies. Therefore, more high-quality studies are needed to fully confirm the effect of adrenaline on the long-term results of OHCA.

## Data Availability

The datasets used and/or analyzed during the current study are available from the corresponding author on reasonable request.

## Abbreviations

OHCA: Out-of-hospital cardiac arrest
CPR: Cardiopulmonary resuscitation
CA: Cardiac arrest
ROSC: Return of spontaneous circulation
RCTs: Randomized controlled trials
IHCA: In-hospital cardiac arrest
VT: Ventricular tachycardia
VF: Ventricular fibrillation
PEA: Pulseless electrical activity

## References

[1] Benjamin EJ, Muntner P, Alonso A, Bittencourt MS, Callaway CW, Carson AP, Chamberlain AM, Chang AR, Cheng S, Das SR (2019). Heart disease and stroke statistics-2019 update: a report from the American Heart Association. Circulation.

[2] Holmberg MJ, Issa MS, Moskowitz A, Morley P, Welsford M, Neumar RW, Paiva EF, Coker A, Hansen CK, Andersen LW (2019). Vasopressors during adult cardiac arrest: a systematic review and meta-analysis. Resuscitation.

[3] Hagihara A, Hasegawa M, Abe T, Nagata T, Wakata Y, Miyazaki S (2012). Prehospital epinephrine use and survival among patients with out-of-hospital cardiac arrest. JAMA.

[4] Callaway CW (2012). Questioning the use of epinephrine to treat cardiac arrest. JAMA.

[5] Callaway CW (2013). Epinephrine for cardiac arrest. Curr Opin Cardiol.

[6] Matsuyama T, Komukai S, Izawa J, Gibo K, Okubo M, Kiyohara K, Kiguchi T, Iwami T, Ohta B, Kitamura T (2022). Epinephrine administration for adult out-of-hospital cardiac arrest patients with refractory shockable rhythm: time-dependent propensity score-sequential matching analysis from a nationwide population-based registry. Eur Heart J Cardiovasc Pharmacother.

[7] Perkins GD, Kenna C, Ji C, Deakin CD, Nolan JP, Quinn T, Scomparin C, Fothergill R, Gunson I, Pocock H (2020). The influence of time to adrenaline administration in the paramedic 2 randomised controlled trial. Intensive Care Med.

[8] Perkins GD, Ji C, Deakin CD, Quinn T, Nolan JP, Scomparin C, Regan S, Long J, Slowther A, Pocock H (2018). A randomized trial of epinephrine in out-of-hospital cardiac arrest. N Engl J Med.

[9] Ong ME, Tan EH, Ng FS, Panchalingham A, Lim SH, Manning PG, Ong VY, Lim SH, Yap S, Tham LP (2007). Survival outcomes with the introduction of intravenous epinephrine in the management of out-of-hospital cardiac arrest. Ann Emerg Med.

[10] Wang HE, Min A, Hostler D, Chang CC, Callaway CW (2005). Differential effects of out-of-hospital interventions on short- and long-term survival after cardiopulmonary arrest. Resuscitation.

[11] Stiell IG, Wells GA, Field B, Spaite DW, Nesbitt LP, De Maio VJ, Nichol G, Cousineau D, Blackburn J, Munkley D (2004). Advanced cardiac life support in out-of-hospital cardiac arrest. N Engl J Med.

[12] Kempton H, Vlok R, Thang C, Melhuish T, White L (2019). Standard dose epinephrine versus placebo in out of hospital cardiac arrest: a systematic review and meta-analysis. Am J Emerg Med.

[13] Vargas M, Buonanno P, Iacovazzo C, Servillo G (2019). Epinephrine for out of hospital cardiac arrest: a systematic review and meta-analysis of randomized controlled trials. Resuscitation.

[14] Ludwin K, Safiejko K, Smereka J, Nadolny K, Cyran M, Yakubtsevich R, Jaguszewski MJ, Filipiak KJ, Szarpak L, Rodríguez-Núñez A (2021). Systematic review and meta-analysis appraising efficacy and safety of adrenaline for adult cardiopulmonary resuscitation. Cardiol J.

[15] Sauneuf B, Dupeyrat J, Souloy X, Leclerc M, Courteille B, Canoville B, Ramakers M, Goddé F, Beygui F, du Cheyron D (2020). The CAHP (cardiac arrest hospital prognosis) score: a tool for risk stratification after out-of-hospital cardiac arrest in elderly patients. Resuscitation.

[16] Machida M, Miura S, Matsuo K, Ishikura H, Saku K (2012). Effect of intravenous adrenaline before arrival at the hospital in out-of-hospital cardiac arrest. J Cardiol.

[17] Kaji AH, Hanif AM, Bosson N, Ostermayer D, Niemann JT (2014). Predictors of neurologic outcome in patients resuscitated from out-of-hospital cardiac arrest using classification and regression tree analysis. Am J Cardiol.

[18] Higgins JPT, Green S. Cochrane handbook for systematic reviews of interventions. Version 5.1.0. The Cochrane Collaboration. 2011.

[19] Kocjancic ST, Jazbec A, Noc M (2014). Impact of intensified postresuscitation treatment on outcome of comatose survivors of out-of-hospital cardiac arrest according to initial rhythm. Resuscitation.

[20] Nordseth T, Olasveengen TM, Kvaløy JT, Wik L, Steen PA, Skogvoll E (2012). Dynamic effects of adrenaline (epinephrine) in out-of-hospital cardiac arrest with initial pulseless electrical activity (PEA). Resuscitation.

[21] Jacobs IG, Finn JC, Jelinek GA, Oxer HF, Thompson PL (2011). Effect of adrenaline on survival in out-of-hospital cardiac arrest: a randomised double-blind placebo-controlled trial. Resuscitation.

[22] Baert V, Hubert H, Chouihed T, Claustre C, Wiel É, Escutnaire J, Jaeger D, Vilhelm C, Segal N, Adnet F (2020). A time-dependent propensity score matching approach to assess epinephrine use on patients survival within out-of-hospital cardiac arrest care. J Emerg Med.

[23] Fukuda T, Fukuda-Ohashi N, Doi K, Matsubara T, Yahagi N (2015). Effective pre-hospital care for out-of-hospital cardiac arrest caused by respiratory disease. Heart Lung Circ.

[24] Fukuda T, Ohashi-Fukuda N, Matsubara T, Gunshin M, Kondo Y, Yahagi N (2016). Effect of prehospital epinephrine on out-of-hospital cardiac arrest: a report from the national out-of-hospital cardiac arrest data registry in Japan, 2011–2012. Eur J Clin Pharmacol.

[25] Neset A, Nordseth T, Kramer-Johansen J, Wik L, Olasveengen TM (2013). Effects of adrenaline on rhythm transitions in out-of-hospital cardiac arrest. Acta Anaesthesiol Scand.

[26] Hayakawa M, Gando S, Mizuno H, Asai Y, Shichinohe Y, Takahashi I, Makise H (2013). Effects of epinephrine administration in out-of-hospital cardiac arrest based on a propensity analysis. J Intensive Care.

[27] Hayashi Y, Iwami T, Kitamura T, Nishiuchi T, Kajino K, Sakai T, Nishiyama C, Nitta M, Hiraide A, Kai T (2012). Impact of early intravenous epinephrine administration on outcomes following out-of-hospital cardiac arrest. Circ J.

[28] Nakahara S, Tomio J, Takahashi H, Ichikawa M, Nishida M, Morimura N, Sakamoto T (2013). Evaluation of pre-hospital administration of adrenaline (epinephrine) by emergency medical services for patients with out of hospital cardiac arrest in Japan: controlled propensity matched retrospective cohort study. BMJ.

[29] Wang Y, Zhang Q, Qu GB, Fang F, Dai XK, Yu LX, Zhang H (2022). Effects of prehospital management in out-of-hospital cardiac arrest: advanced airway and adrenaline administration. BMC Health Serv Res.

[30] Dumas F, Bougouin W, Geri G, Lamhaut L, Bougle A, Daviaud F, Morichau-Beauchant T, Rosencher J, Marijon E, Carli P (2014). Is epinephrine during cardiac arrest associated with worse outcomes in resuscitated patients?. J Am Coll Cardiol.

[31] Olasveengen TM, Wik L, Sunde K, Steen PA (2012). Outcome when adrenaline (epinephrine) was actually given vs. not given–hoc analysis of a randomized clinical trial. Resuscitation.

[32] Goto Y, Maeda T, Goto Y (2013). Effects of prehospital epinephrine during out-of-hospital cardiac arrest with initial non-shockable rhythm: an observational cohort study. Crit Care.

[33] Ornato JP (2008). Optimal vasopressor drug therapy during resuscitation. Crit Care.

[34] Michael JR, Guerci AD, Koehler RC, Shi AY, Tsitlik J, Chandra N, Niedermeyer E, Rogers MC, Traystman RJ, Weisfeldt ML (1984). Mechanisms by which epinephrine augments cerebral and myocardial perfusion during cardiopulmonary resuscitation in dogs. Circulation.

[35] Loomba RS, Nijhawan K, Aggarwal S, Arora RR (2015). Increased return of spontaneous circulation at the expense of neurologic outcomes: is prehospital epinephrine for out-of-hospital cardiac arrest really worth it?. J Crit Care.

[36] Morales-Cané I, Valverde-León MD, Rodríguez-Borrego MA (2016). Epinephrine in cardiac arrest: systematic review and meta-analysis. Rev Lat Am Enfermagem.

[37] Lin S, Callaway CW, Shah PS, Wagner JD, Beyene J, Ziegler CP, Morrison LJ (2014). Adrenaline for out-of-hospital cardiac arrest resuscitation: a systematic review and meta-analysis of randomized controlled trials. Resuscitation.

[38] Perkins GD, Cottrell P, Gates S (2014). Is adrenaline safe and effective as a treatment for out of hospital cardiac arrest?. BMJ.

[39] Larsson PT, Wallén NH, Egberg N, Hjemdahl P (1992). Alpha-adrenoceptor blockade by phentolamine inhibits adrenaline-induced platelet activation in vivo without affecting resting measurements. Clin Sci.

[40] Ristagno G, Tang W, Huang L, Fymat A, Chang YT, Sun S, Castillo C, Weil MH (2009). Epinephrine reduces cerebral perfusion during cardiopulmonary resuscitation. Crit Care Med.

[41] Casas AI, Geuss E, Kleikers PWM, Mencl S, Herrmann AM, Buendia I, Egea J, Meuth SG, Lopez MG, Kleinschnitz C (2017). NOX4-dependent neuronal autotoxicity and BBB breakdown explain the superior sensitivity of the brain to ischemic damage. Proc Natl Acad Sci USA.

[42] Panchal AR, Bartos JA, Cabañas JG, Donnino MW, Drennan IR, Hirsch KG, Kudenchuk PJ, Kurz MC, Lavonas EJ, Morley PT (2020). Part 3: adult basic and advanced life support: 2020 American Heart Association guidelines for cardiopulmonary resuscitation and emergency cardiovascular care. Circulation.

[43] Arrich J, Sterz F, Herkner H, Testori C, Behringer W (2012). Total epinephrine dose during asystole and pulseless electrical activity cardiac arrests is associated with unfavourable functional outcome and increased in-hospital mortality. Resuscitation.

[44] Fried TR, Bradley EH, Towle VR (2002). Assessment of patient preferences: integrating treatments and outcomes. J Gerontol B Psychol Sci Soc Sci.

[45] Deakin CD, Nolan JP, Soar J, Sunde K, Koster RW, Smith GB, Perkins GD (2010). European Resuscitation Council Guidelines for Resuscitation Council Guidelines for Resuscitation 2010 Section 4. Adult advanced life support. Resuscitation.

[46] Peberdy MA, Callaway CW, Neumar RW, Geocadin RG, Zimmerman JL, Donnino M, Gabrielli A, Silvers SM, Zaritsky AL, Merchant R (2010). Part 9: post-cardiac arrest care: 2010 American Heart Association guidelines for cardiopulmonary resuscitation and emergency cardiovascular care. Circulation.

[47] Andersen LW, Kurth T, Chase M, Berg KM, Cocchi MN, Callaway C, Donnino MW (2016). Early administration of epinephrine (adrenaline) in patients with cardiac arrest with initial shockable rhythm in hospital: propensity score matched analysis. BMJ.

